# Validated UPLC-MS/MS method for the quantification of dasatinib in plasma: Application to pharmacokinetic interaction studies with nutraceuticals in Wistar rats

**DOI:** 10.1371/journal.pone.0199208

**Published:** 2018-06-14

**Authors:** Hadir M. Maher, Nourah Z. Alzoman, Shereen M. Shehata, Norah O. Abanmy

**Affiliations:** 1 College of Pharmacy, Department of Pharmaceutical Chemistry, King Saud University, Riyadh, Saudi Arabia; 2 Faculty of Pharmacy, Department of Pharmaceutical Analytical Chemistry, University of Alexandria, El-Messalah, Alexandria, Egypt; 3 College of Pharmacy, Department of Clinical Pharmacy, King Saud University, Riyadh, Saudi Arabia; Tokyo University of Agriculture, JAPAN

## Abstract

Dasatinib (DAS) is a tyrosine kinase inhibitor (TKI) used in the treatment of chronic myeloid leukemia and in the management of ulcerative colitis (UC). Since some nutraceuticals (e.g. curcumin, olive oil, and cocoa extract) could alter the function of ABC transporters and /or CYP450 enzymes, DAS bioavailability could potentially be affected following their co-administration. This work aims at studying the possibility of PK interaction between DAS and the selected nutraceuticals in UC rats using UPLC- MS/MS. Chromatographic analysis was carried out using BEH C 18 column (Waters) with a mobile phase consisting of acetonitrile and 50% aqueous methanol, 65:35, v/v, each with 0.1% formic acid and using erlotinib (ERL) as an internal standard (IS). DAS quantitation was carried out using multiple reaction monitoring (MRM) with positive ionization of the transitions at *m/z* 488.03 > 400.92 (DAS), and *m/z* 394.29 > 278.19 (ERL). Method validation was assessed as per the FDA guidelines for bioanalytical methods for DAS determination within the concentration range 1–500 ng/mL. No significant effect on the oral bioavailability of DAS was reported with any of the studied nutraceuticals. Thus, the concomitant administration of these nutraceuticals with DAS could be considered safe with a necessity to perform more detailed clinical investigations.

## Introduction

Dasatinib (DAS) is a second generation tyrosine kinase inhibitor (TKI) prescribed mainly for the treatment of patients with imatinib-resistant or intolerant chronic myeloid leukemia (CML). DAS has also been approved for the management of Philadelphia chromosome (Ph)-positive acute lymphoblastic leukemia (ALL) [1]. In spite of the fact that DAS is particularly known for its anticancer effect, it is suggested that DAS could decrease colon inflammation and is thus proposed as a treatment strategy in patients with ulcerative colitis (UC) [2]. Since many inflammatory diseases are associated with hyperactivity in tyrosine kinases, TKIs could be beneficial in this respect. In addition, TKIs suppress the synthesis of pro-inflammatory cytokines including tumor necrosis factor alpha (TNFα) and interleukins 1 and 6 (IL-1, IL-6) [2]. In addition, DAS is known for its inhibitory effect on Scr tyrosine kinase (SFK) showing an extra suppression of angiogenesis, proliferation, and survival of cancer cells, as well as decreasing vascular permeability. This SKF inhibitory action of DAS contributes to its beneficial effect in the treatment of UC since the latter is associated with increased vascular permeability and Scr kinase activity [3].

Being oral drugs, TKIs are subjected to large intra-individual and inter-individual pharmacokinetic (PK) variability [4]. Generally, the bioavailability of TKIs is mainly affected by the extent of drug absorption and metabolism. DAS is absorbed through carrier-mediated transport controlled by the ATP binding cassette (ABC) transporters, mainly permeability glycoprotein (P-gp) and the breast cancer resistance protein (BCRP), in addition to the solute carrier (SLC) transporters, particularly organic anion-transporting polypeptide (OATP) [4–6]. TKIs, including DAS, are metabolized mainly by cytochrome P450 (CYP450) enzymes that are responsible of phase I oxidative metabolism of most drugs in the intestine and liver [4–6]. Plasma levels of TKIs are thus largely affected by the function of membrane transporters as well as CYP450 metabolizing enzymes. The large intra- and inter-variability in the PK of TKIs could be attributed to the heterogeneity in the pharmacogenetics (e.g. polymorphism in CYP450 or transporters), genetic heterogeneity of drug targets, patient-adherence to treatment, in addition to environmental factors (e.g. smoking and drug/drug interactions) [1, 4–6]. Alteration in the exposure to the active drug may lead to altered treatment efficiency or increased toxicity. Recently, drug interactions between TKIs and co-administered drugs, food, herbal medicine, and beverages are a matter of special concern. PK interaction of DAS has been previously referred to acid-suppression (e.g. famotidine, omeprazole), CYP3A4 induction (e.g. rifampicin), and CYP3A4 inhibition (e.g. ketoconazole) [6]. Also, the interaction of fruit juices with DAS through inhibition of BCRP has been reported [7]. It is also important to mention that the issue of food-drug interactions, including those between TKIs and herbs, has recently become a major concern. This could be attributed to the growing use of herbs as complementary medicine, most of which has been marketed as nutraceuticals. In this study, a special concern would be paid to curcumin, olive oil, and cocoa extract as vital nutraceuticals that could possibly be co-administered with DAS.

Curcumin is a natural phenolic compound extracted from *Curcuma Longa*, known as turmeric, widely used in traditional medicine as a spice, as well as an efficient medicinal plant. Recently, curcumin has gained much attention for its use as a potential adjuvant in the treatment of cancerous patients. It has been known to have antioxidant properties, as well as inhibitory effect of hydrogen peroxide-induced cell damage. Moreover, the anti-inflammatory, anti-HIV, anti-depressant, anticonvulsant, antimicrobial, antifungal, anti-rheumatic properties of curcumin have been documented [8, 9]. Clinical studies have suggested the synergistic effect of curcumin with DAS in inhibiting colon cancer [10]. Thus curcumin and DAS could be used as a combination therapy for the treatment of cancerous patients. However, the possible effect of curcumin on the PK and hence the bioavailability of DAS has not been studied till now. Given its safety, high tolerability, and wide consumption in many populations, drug interactions with curcumin is a matter of public concern. This could be attributed to its inhibitory effect on the expression of P-gp transporters, as well as the CYP3A activity [11–13]. Many curcumin-mediated drug interactions have been reported; curcumin increased the bioavailability of docetaxel, etoposide, midazolam, peroral, and celiprolol in rats [11–13].

Olive oil, extracted from *Olea Europea L*., is one of the most well-known dietary components with health benefits. Extra virgin olive oil (EVOO) is obtained from the fruits of the olive tree by mechanical or physical methods that do not cause oil alteration [14–16]. Overview of the most bioactive effects of EVOO reveals its cardiovascular-protective, anti-inflammatory, joint-protective, anticancer, hepato-protective, antidiabetic, and anti-HIV effects [14–17]. The anticancer effect of the EVOO is mainly related to its antioxidant, anti-proliferative, anti-apoptotic, and immunomodulatory action [14]. These health protective effects are attributed not only to the high monounsaturated fatty acid content, but also to the minor bioactive phenolic compounds, the most active of which are hydroxyl tyrosol, tyrosol, and oleuropein [14]. EVOO-rich diet was confirmed to have a protective effect in UC-associated colorectal cancer (CRC), acute colitis, and chronic colitis [15, 18, 19]. In this respect, it is important to mention that olive oil triterpenoids are reported to have inhibitory effect on CYP450 enzymes, particularly CYP1A and CYP2A [16]. Another study reported the inhibitory effect of maslinic acid, a bioactive compound in olive fruit, on CYP450 activity in both humans (CYP3A4) and rats (CYP2C11, CYP3A2) [19]. Giving the curative effect of both DAS and EVOO in CRC and different types of colitis, besides their chemo-preventive effect, their concomitant administration is likely to be effective in this respect. Yet, the inhibitory effect of EVOO on the CYP340 activity, suggests that special attention should be paid with its use as a nutraceutical with DAS.

Cocoa, a product of *Theobroma cacao*, has widely gained much attention due to its diverse biological benefits [20–25]. Cocoa is enriched with two classes of bioactive compounds, namely polyphenols and alkaloids. Among the most active cocoa polyphenols are catechin, epicatechin, quercetin, epigallocatechin-3-gallate, and procyanidins [21]. Cocoa flavonoids have many documented biological activities, e.g. cardio-protective, immune-stimulant, anti-inflammatory, anticancer, anti-diabetic, and hepato-protective effects [21–23, 25]. In addition, polyphenol-enriched cocoa extract was found beneficial in the management of UC [24]. It has also been reported that increased cocoa consumption is associated with decreased liver enzymes in HIV-HCV co-infected patients [26]. The role of cocoa polyphenols in the management of psychiatric disorders and modulation of mental health has also gained much interest [27]. Moreover, cocoa polyphenols are reported to attenuate hyper-caloric diet-induced obesity and exert positive effects on obesity-related metabolic risk factors [28]. Concomitant administration of DAS and cocoa extract not only has synergistic anticancer effect, but also shows additive protective effect in the management of UC. Since polyphenolic compounds are reported to inhibit CYP3A, cocoa extract could possibly alter the bioavailability of DAS.

In spite of the widespread of nutraceuticals, namely curcumin, olive oil, cocoa extract, and their reported inhibitory effect on the CYP enzymes, their possible PK interaction with TKIs have not been studied yet. Since DAS could be used concomitantly with any of the above mentioned nutraceuticals for their synergistic effect in the management of UC or for their anticancer effect, it is important to study their possible effect on the bioavailability of DAS. Thus it was thought to mimic the actual situation by using rats with UC. Because of its high sensitivity and selectivity, LC- MS/MS method was the method of choice for measuring DAS in plasma levels [29–31]. This work aims at developing and validation of UPLC -MS/MS method for the purpose of monitoring the PK interaction between DAS and each of the above-mentioned nutraceuticals in acetic acid-induced UC rats. This is extremely beneficial with TDM of DAS, for the purpose of dose individualization to get the required therapeutic outcome with minimum side effects.

## Materials and methods

### Chemicals and reagents

Reference standards of DAS (purity >99%) and erlotinib (ERL) internal standard (IS) (purity >99%) were purchased from Haoyuan Chemexpress Co., Ltd., Shanghai, P.R. China. The solvents involved in the study were methanol and acetonitrile (Panreac, E.U.). Also, formic acid (Sigma Aldrich, Chemie GmbH, Steinheim, Germany) was used. Different nutraceuticals of curcumin, olive oil, and cocoa were involved in the study. Two curcumin preparations were purchased, namely Super Bio-curcumin^®^ vegetarian capsules, curcumin I, (Life Extension^®^, Quality Supplements and Vitamins, Inc., Florida, USA), labeled to contain 400 mg turmeric root extract, and Curcumin 95 veggie capsules, curcumin II (Jarrow formulas^®^, Super Nutrition and Formulation^SM^, Los Angeles, CA, USA), labeled to contain 500 mg turmeric concentrate/capsule. The sources of olive oil employed in the study were, Olive Leaf Extract 500 mg vegetarian capsules, olive oil I (NOW FOODS^®^, Bloomingdale, IL, USA), labeled to contain 500 mg of olive leaf extract per capsule, standardized to min. 6% oleuropein, and EVOO, olive oil II (Bionaturae^®^, Euro-USA Trading Co. Inc., North Franklin, CT). To investigate the effect of cocoa on the PK of DAS, two CocoaVia^®^ (®/™ Trademarks Mars, Inc., Germantown, MD, USA) brand products were applied, vegetarian capsules, cocoa I, labeled to contain 1350 mg cocoa bean extract equivalent to 375 mg cocoa flavanols per serving size of three capsules, and unsweetened dark chocolate mix packets, cocoa II, labeled to contain 1500 mg cocoa bean extract equivalent to 375 mg cocoa flavanols. Ultrapure water involved in the study was obtained from Ultrapure water Milli-Q Advantage water purification system, 0.22μm filter (Millipore, Molsheim, France).

### Instrumentation and analytical conditions

UPLC–MS/MS Waters Model Xevo TQ-S separation system (Waters, Singapore, Singapore) was employed in the study. The instrument was equipped with binary solvent manager and sample manager (Aquity^TM^ Ultra-performance LC). Detection was performed using triple-quadrupole mass spectrometer (STEP WAVE^TM^, Ultra-performance LC) with electrospray ion source (Zspray^TM^ ESI-APCI-ESCI, Ultra-performance LC), in addition to multiple reaction monitoring (MRM). The output data was processed using Masslynx^TM^ Version 4.1 software (Micromass, Manchester, UK).

Chromatographic separation was carried out using Acquity UPLC BEH^TM^ C 18 analytical column (Waters, Dublin, Ireland), with dimensions 100 × 1.0 mm, i.d., 1.7 μm particle size. The mobile phase used consisted of acetonitrile and 50% aqueous methanol, 65:35, v/v, each with 0.1% formic acid. Volumes of 5 μL were separately injected into the column using the partial loop mode at the flow rate of 0.2 mL/min. Temperatures of the auto-sampler and column were kept at 4 ^o^ and 45 ^o^ C, respectively. Sample preparation was carried out using solid-phase extraction (SPE) with Strata^®^ C 18-E (55μm, 70 A^o^) (200 mg, 3 mL) tubes (Phenomenex Inc., Torrance, CA, USA). Nitrogen evaporator N-EVAP 112 supplied with heating system OA-SYS (Organomation Assocciates, Inc, MA, USA) and Bakers vacuum system were used. Disposable syringe filters (CHROMAFIL^®^ Xtra PA-20/25 polyamide filters, 0.2 μm pore size), (MACHEREY NAGEL, GmbH & Co. KG, Duren, Germany), were used for sample preparation.

Mass spectrometric detection was carried out using positive ion electrospray ionization (ESI) source. The employed MS parameters were; desolvation gas flow of 800 L/h, cone gas flow of 150 L/h, collision gas flow of 0.15 mL/min, a source temperature of 150°C, and a dwell time of 0.025 s. MS analyzer was operated using low mass (LM) and high mass (HM) resolutions of 2.8 and 14.86, respectively. Quantitation was performed using MRM of the transitions from protonated molecular ions [M+H]^+^ as precursor ions to product ions, at *m/z* 488.03 > 400.92 (DAS) and *m/z* 394.29 > 278.19 (ERL).

### Stock solutions, calibration standards, and quality control (QC) samples

Stock solutions of DAS and ERL (IS) at 1 mg/mL were prepared using methanol. Further dilutions were made using the same solvent to get standard solutions of different concentrations of DAS. Also, a standard solution of 50 ng/mL of ERL was prepared.

Eight calibration standards were prepared by spiking separate 50 μL volumes of blank plasma samples with standard solutions of DAS, such that final DAS concentrations were within the working range of 1–500 ng/mL, along with 50 μL of 50 ng/mL ERL (IS). Spiked samples were then made up to final volumes of 1 mL with methanol. Blank plasma samples were prepared simply by mixing 50 μL plasma with 950 μL methanol. Similar to calibration standards, quality control (QC) samples, used for the validation purpose, were prepared at four different concentration levels of DAS, namely 1, 3, 250, and 450 ng/mL for very low (LLOQ), low, medium, and high concentration levels, respectively, along with ERL (IS).

### Sample preparation

Calibration standards, QC samples, as well as blank plasma samples were centrifugated at 6000 rpm for 5 min. Following the separation of the methanolic supernatants, the residues of individual samples were separately washed with 0.5 mL volumes of methanol. The combined supernatant and washing of each sample were cleaned-up by passing onto STRATA^®^ C 18-E (200 mg, 3 mL) SPE cartridges, previously preconditioned with 3.0 mL methanol followed by 3.0 mL ultrapure water. The retained drugs were eluted from each sample using separate volumes of 0.5 mL methanol. Following the evaporation of the eluted methanolic solutions to dryness under nitrogen, the residue was reconstituted in 0.5 mL acetonitrile. Finally, UPLC–MS/MS analysis of the reconstituted samples was performed by injecting 5 μL volumes of each sample under the operating conditions.

### Assay validation

#### Specificity

The specificity of the method was evaluated by comparing the chromatograms of plasma samples spiked with DAS at its LLOQ concentration level with those of blank plasma samples obtained from six different rats, along with ERL (IS). Peak area ratios obtained at DAS retention time in each case were compared in order to ensure the absence of interference at the elution time of the studied drug, DAS.

#### Linearity

Method linearity was evaluated by spiking different volumes (50 μL) of drug-free plasma samples with eight different concentrations of DAS in the range 1–500 ng/mL plasma, along with 50 μL of 50 ng/mL ERL (IS). Following the analysis of each sample, the peak area ratios of DAS to that of ERL (IS) were related to the spiked DAS concentrations to get the matrix-based calibration graph and the corresponding regression equation.

#### Lower limit of detection (LLOD) and lower limit of quantification (LLOQ)

LLOD and LLOQ of DAS were calculated based on DAS concentrations that produced analytical responses of at least three times or five times that of the blank signal, respectively. Moreover, for LLOQ, the signals produced should be well-identified with at least 20% errors and relative standard deviations (RSD), assessing acceptable degree of accuracy and precision, respectively.

#### Extraction recovery and matrix effect

The extraction recovery of DAS from plasma samples was assessed at the four different QC levels, very low LLOQ (1 ng/mL), low (3 ng/mL), medium (250 ng/mL), and high (450 ng/mL). This was achieved by comparing the peak area obtained from plasma samples spiked pre-extraction with those spiked post-extraction with the same nominal concentration level (n = 6). Moreover, the extraction recovery of ERL (IS) was also determined at the same concentration level used in actual analysis. Matrix effect was evaluated at the same QC levels of DAS as those used in evaluating the extraction recovery. However, the peak area obtained for DAS following the analysis of samples spiked post-extraction were compared with those of standard DAS prepared directly in acetonitrile and having the same concentrations. Similarly, the matrix effect of ERL (IS), at the same concentration level used in the analysis, was calculated.

#### Precision and accuracy

Accuracy and precision were evaluated at two levels, intra-day, on the same day (n = 6), and inter-day, on three consecutive days (n = 18). This was performed by analyzing the four QC samples prepared at LLOQ, low, medium, and high DAS concentration levels. For each solution, DAS peak area ratio to that of ERL (IS) was used to calculate the actual DAS concentration by substitution into the regression equation and then compared with the nominal values. Finally, percentage relative error (E_r_%) and percentage relative standard deviation (%RSD) were calculated to assess the accuracy and precision, respectively.

#### Dilution integrity

Dilution of highly concentrated plasma samples, with concentrations beyond the linearity range of the proposed method, was evaluated for its effect on DAS recoveries. Plasma samples spiked with high concentrations of DAS (800 ng/mL) were used following dilution with blank plasma samples, dilution folds (1:2 and 1:5). Diluted samples were then treated as under "Sample preparation”. Following actual analysis, the found concentrations were calculated for each sample and then related to the nominal values to calculate the % recovery.

#### Stability studies

QC samples spiked at two concentration levels of DAS, low (3 ng/mL) and high (450 ng/mL), were analyzed (n = 6) in order to assess the drug stability in plasma. Stability testing were performed by exposing the QC plasma samples to different conditions, namely auto-sampler stability (extracted samples left in the auto-sampler at 10°C for 56 h before injection), short-term (bench-top) stability (samples left at room temperature (25 ^o^ C) for 6 h), and long-term stability (samples left at -30°C for 30 days). Moreover, freeze-thaw stability was assessed as follows, plasma samples were frozen at around -30°C and then thawed at room temperature for three cycles. For each sample, DAS concentrations were related to the nominal concentrations to calculate the % recovery.

### Ethics statement

The study protocol was approved by the local Research Ethics committee of Animal Experiments, King Saud University, Riyadh, Saudi Arabia. All experiments were carried out in accordance with the ethical guidelines for experimental studies with animals as per the local Research Ethics committee, King Saud University.

### Application to pharmacokinetic studies

Wistar healthy male rats weighing 250 ± 30 g were provided by the animal house, Women Student-Medical studies & Sciences Sections, College of Pharmacy, King Saud University, Riyadh, Saudi Arabia. The rats were placed in cages kept in a well-ventilated room and subjected to a regular 12 h day–night cycle at a relative humidity of 40–60% and average temperature of 24–27°C. All the rats could access the water freely while diet was prohibited for 12 h before drug administration. The rats were acclimatized for 7 days to laboratory conditions before conducting the experiment. Seven groups of five rats each were involved in the study. For all rats, UC was induced by oral daily administration of 1 mL volumes of 5% glacial acetic acid in water for three successive days. DAS suspension was prepared by triturating 125 mg of DAS with 5 mL aqueous methyl cellulose (0.5%, w/v) to prepare DAS suspension of 25 mg/mL, given to all groups with the exception of group V where olive oil was used for the suspension preparation. Curcumin preparations (50 mg/mL) were prepared by suspending the content of one capsule of either Curcumin 95 500 mg or Super Bio-Curcumin 400 mg in 10 mL or 8 mL volumes of water, respectively. By analogy, a suspension of 50 mg/mL olive leaf extract was prepared by suspending the content of one capsule into 10 mL water. However, for cocoa preparations, 450 mg cocoa extract/ 5 mL, aqueous suspensions of either CocoaVia^®^ capsules or CocoaVia^®^ unsweetened dark chocolate powder were prepared by suspending the content of one capsule or 2 g powder, respectively in water. Separate volumes (0.25 mL) of DAS suspension were orally administered to all treated animals using a gavage needle so that all rats received DAS dose of 25 mg/kg. For the purpose of comparison, rats of group I were only treated with DAS. Rats of groups II and III, curcumin groups, were first separately given an oral dose of 50 mg/mL of either Super Bio-Curcumin, curcumin I (rat group II) or Curcumin 95, curcumin II (rat group III). Then 30 min following curcumin administration, DAS suspension was given to the rats in a dose of 25 mg/kg. Rats of groups IV and V, the olive oil groups, were administered olive oil as follows; rats of group IV were separately given an oral dose of olive leaf extract, olive oil I, 50 mg/kg 30 min prior to DAS administration (25 mg/kg), while rats of groups V were separately given an oral dose of DAS suspended in olive oil, olive oil II (25 mg/kg). Finally, rats of groups VI and VII, cocoa groups, were initially given an oral dose of CocoaVia^®^ capsules, cocoa I, 90 mg/kg (rat group VI), or CocoaVia^®^ unsweetened dark chocolate, cocoa II, 90 mg/kg (rat group VII) 30 min before DAS dosing of 25 mg/kg. For each group, volumes of 0.3 mL blood samples were withdrawn from the retro-orbital sinus of each rat into heparinized tubes. Blood samples were collected at different time intervals; 0 (prior to dosing), 0.5, 1, 2, 3, 5, 6, and 24 h following DAS administration. All collected blood samples were centrifuged immediately at 4,500 rpm (30 min, 4°C). Plasma samples obtained after centrifugation were kept frozen at -20°C till the day of analysis. From each plasma sample, volumes of 50 μL were separately spiked with ERL as IS, 50 μL of 50 ng/mL ERL. Spiked samples were then completed to a final volume of 1 mL with methanol and were then treated exactly as described under sample preparation.

### Pharmacokinetic calculations

PKSolver 2.0 Add-in Excel 2010 was used to process the DAS plasma concentrations as a function of the analysis time. For all groups, PK parameters were calculated based on a non-compartmental analysis technique (NCA). Statistical significance was tested between the control group (I) and the treated groups (II to VII) using Dunnett’s test with ANOVA at α = 0.05.

## Results and discussion

### Optimization of UPLC-MS/MS conditions

**Fig 1:**
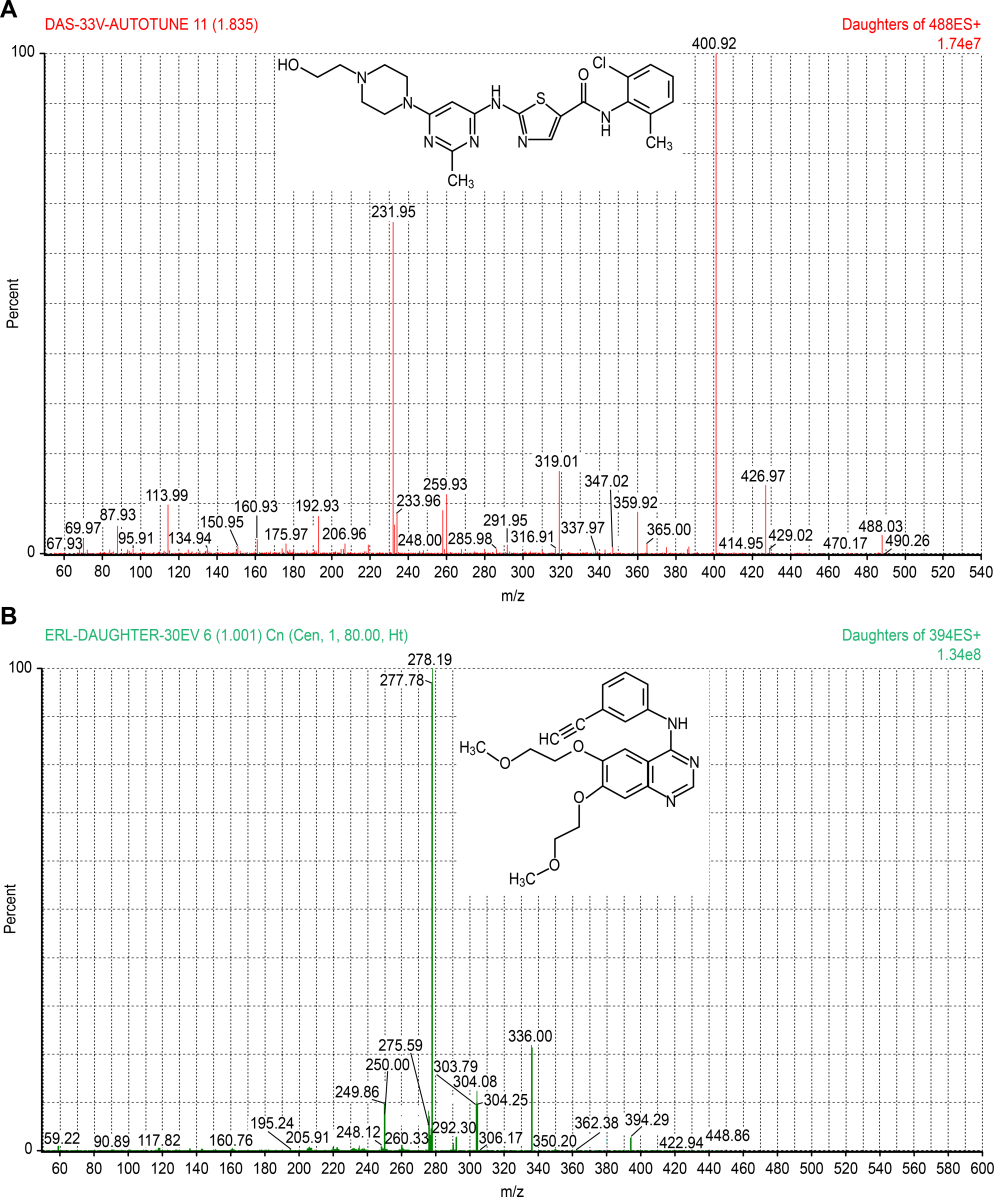
**Product ion spectra of DAS, a) and ERL, b)**.

Both chromatographic and mass spectrometric conditions were optimized for getting highly sensitive and selective analysis. Initially, MS/MS conditions were optimized by syringe infusion of standard solutions of DAS and ERL (IS), Fig 1, (1 ng/mL) into the mass spectrometer. Efficient ionization, with higher sensitivity and selectivity, was recorded for both DAS and ERL using the positive ionization mode relative to the negative ionization mode. Thus, MRM with positive ESI was used in order to monitor the precursor as well as the product ions. Precursor ions [M+H]^+^ were monitored at *m/z* 488.03 (DAS) and *m/z* 394.29 (ERL), while the product ions were at *m/z* 400.92 (DAS) and *m/z* 278.19 (ERL). For highest intensity of the protonated molecular ions, different MS/MS parameters were adjusted as follows: a source temperature of 150 ^o^C, desolvation gas flow rate of 80 L/h, cone voltage of 10 (DAS) and 25 V (ERL), capillary voltage of 3.8 (DAS) and 3.5 KV (ERL), and a desolvation temperature of 200^o^ C for both compounds. However, for maximum intensity of the selected product ions, the collision energy was optimized to 33 (DAS) and 30 eV (ERL). The optimized mass spectrometric conditions of both DAS and ERL were summarized in Table 1. Full scan product ion spectra of protonated molecular ions for DAS and ERL were shown in Fig 1.

**Table 1:** LC–MS/MS optimized parameters for the determination of the studied drugs.

Target compound	Precursor ion [M+H]^+^	Daughter ion	Cone voltage (V)	Capillary voltage (KV)	Collision energy (eV)	Desolvation Temperature (^o^ C)
DAS	488.03	400.92	10	3.8	33	200
ERL (IS)	394.29	278.19	25	3.5	30	200

The second phase of the optimization procedure involves investigating the effect of mobile phase composition on the chromatographic behavior of both DAS and ERL. In this respect, mixtures of different ratios of acetonitrile (30–90%), water, and formic acid (0.05–0.15%) were used as the mobile phase. At first, mobile phases consisting of different ratios of acetonitrile (30–90%) and water, each with 0.1% formic acid, were investigated for their effect on the peak shape and response. Slight tailing in DAS peaks was observed with all ratios of acetonitrile suggesting the incorporation of methanol in further experimentation. It was noticed that peak sharpness and symmetry, along with rapid elution, was recorded for DAS and ERL (IS) with acetonitrile and 50% aqueous methanol, 65: 35, v/v. Thus the latter mobile phase with different ratios of formic acid (0.05–0.15%) was further investigated. Practical experiments showed that the sharpness of DAS peaks was associated with the inclusion of formic acid in the mobile phase and that decreased retention of DAS was noticed with increased formic acid content in the mobile phase till 0.1% above which slight tailing was recorded with DAS peaks. Accordingly, final analysis was carried out using a mobile phase consisting of acetonitrile: 50% aqueous methanol, (65: 35, v/v), each with 0.1% formic acid for the whole runtime of 2 min. Since ERL showed a comparable chromatographic behavior to DAS, it was applied as the IS in this study, thus providing a cheaper alternative to deuterated internal standards. Under the above optimized chromatographic conditions, sharp and symmetric peaks were recorded (DAS eluted at 0.54 ± 0.02 min, and ERL (IS) at 0.49 ± 0.01 min).

### Sample preparation

One of the most critical parameters in the development of bioanalytical techniques is sample preparation. In order to ensure high degree of method selectivity, it is important to get rid of the interfering endogenous sample components as far as possible. SPE has been proven to have a great capacity to eliminate most endogenous biological components. This contributes to its wide applicability in LC-MS/MS biological analysis where ion suppression is one of the main problems arising from the interfering endogenous bio-components. Moreover, a combination of two sample clean up methodologies namely, protein precipitation (PPT) prior to the solid-phase extraction (SPE) guarantees higher grade of interference elimination [32–34] and it was thus applied for sample preparation in this work.

Plasma samples were first treated with methanol for PPT followed by further purification of the clear supernatants using SPE. Initially, two types of SPE cartridges namely, Strata^®^ C 18-E (55μm, 70 Å) (200 mg/3mL) and octyl C 8 (200 mg, 3 mL) were evaluated for their extraction efficiency using plasma samples spiked with DAS at the concentration level of 150 ng/mL. Practical experimentation revealed that better peak shape was obtained with C 18 compared with C 8 cartridges since the latter produced a significant distortion of DAS peaks. Plasma samples spiked with DAS at the QC levels 1, 3, 250, and 450 ng/mL were further used to investigate the excellent extraction recovery of the selected C 18 cartridges for DAS (95.49–98.18%).

### Method validation

Method validation was assessed as per the FDA guidelines for bioanalytical methods [35]. According to the FDA guidelines, QC samples representing the entire range of the standard curve were used for the validation purpose; LLOQ, low QC sample (within 3 times LLOQ), middle QC sample (near the center of the calibration range), and high QC sample (near the upper limit of the calibration range). Different parameters were validated including, method specificity, linearity, lower limits of detection and of quantification, extraction recovery, matrix effect, accuracy and precision, dilution integrity, and stability studies.

#### Specificity

**Fig 2:**
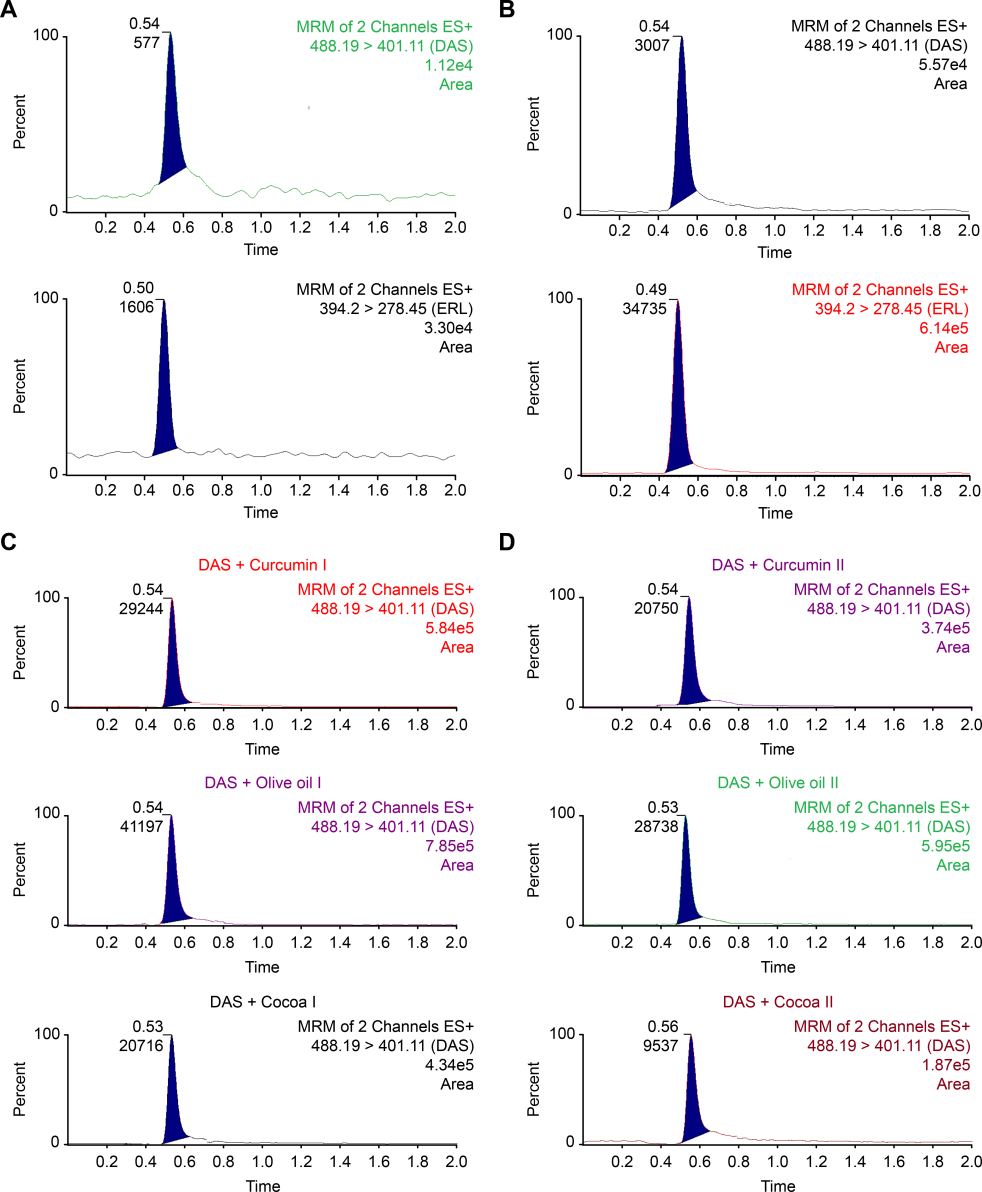
**Multiple reaction monitoring (MRM) of a blank plasma, a), a plasma sample spiked with a standard mixture of DAS at its LLOQ level with ERL (IS), b), and of rat plasma samples 1h following the administration of DAS (25 mg/kg) alone or when co-administered with either curcumin preparations I, II, olive oil, I, II, or cocoa extract preparations I, II, c)**.

The chromatograms of drug-free plasma samples and plasma samples spiked with DAS at its LLOQ level were shown in Fig 2. The peak response of DAS at its LLOQ was at least five times more than that of the blank, while the IS provided peak areas of at least twenty times that of the blank signal. The absence of any interference at the retention times of both DAS and ERL (IS) indicates high degree of method specificity.

#### Linearity

Using the method of least squares, the peak area ratios obtained for DAS to that of ERL (IS) were linearly correlated to the corresponding DAS spiked concentrations in the range 1–500 ng/mL plasma. Accordingly, the regression equation for DAS was derived. Intercept (a), slope (b), and the correlation coefficient (r) were calculated. High degree of method linearity was proven by the small intercept, along with the high value of the correlation coefficient (r = 0.9998). In addition, other statistical parameters were calculated as summarized in Table 2. They included the variance ratio (F values), as well as standard deviations of the intercept (S_a_), of the slope (S_b_), and of residuals (S_y/x_). High F values along with low values of S_y/x_ indicate low degree of scatter of the experimental data points around the regression line [36].

**Table 2:** Regression and statistical parameters for the determination of DAS in rat plasma by the proposed UPLC-MS/MS method.

Linearity range (ng/mL)	1–500
LLOQ (ng/mL)	1.0
LLOD (ng/mL)	0.4
Intercept (a)	-0.0240
Slope (b)	0.0060
Correlation Coefficient (r)	0.9998
S_a_	0.0060
S_b_	0.0005
S_y/x_	0.0169
F	29265.89
Significance F	2.6914×10^−12^

LLOQ, lower limit of quantification; LLOD, lower limit of detection

S_a_, standard deviation of intercept; S_b_, standard deviation of slope

S_y/x_, standard deviation of residuals; F: variance ratio equals the mean of squares due to regression divided by the mean of squares about regression (due to residuals).

#### Lower limit of detection (LLOD) and lower limit of quantification (LLOQ)

**Fig 3:**
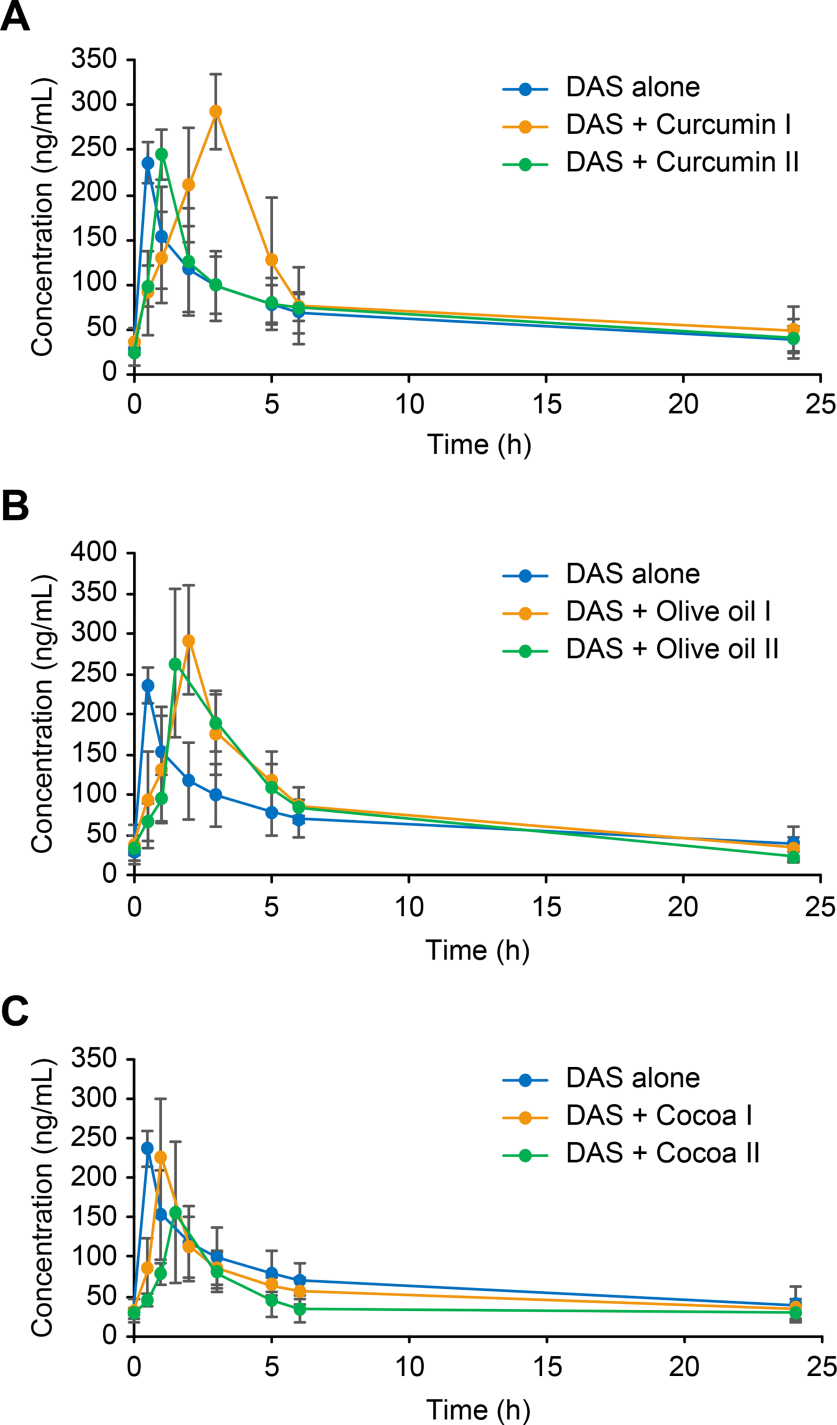
**Plasma concentration–time profile of DAS in rats after an oral administration of a combination of DAS (25 mg/kg) following the administration of DAS (25 mg/kg) alone or when co-administered with either curcumin preparations I, II, a), olive oil, I, II, b), or cocoa extract preparations I, II, c)**.

LLOD and LLOQ for the determination of DAS in plasma using the proposed method were found to be 0.4 and 1 ng/mL, respectively, as mentioned in Table 2. Fig 3 shows the MRM chromatograms of plasma samples spiked with DAS at its LLOQ, along with blank plasma samples. The low value of LLOQ obtained for DAS ensures the applicability of the method for the determination of trace concentrations in plasma, an important issue in TDM and PK studies. The LLOQ obtained for DAS using the proposed method is lower than those obtained using the previously published LC-MS/MS methods for DAS determination [29–31].

#### Recovery and matrix effect

Extraction recovery of DAS from plasma samples were evaluated using QC plasma samples spiked with DAS at different concentration levels, very low LLOQ (1 ng/mL), low (3 ng/mL), medium (250 ng/mL), and high (450 ng/mL). Also, the extraction recovery of ERL (IS) was also evaluated at the concentration level used in actual analysis. Recovery values of not less than 95.11 for DAS and 93.88 for ERL indicate high degree of extraction recovery of the optimized method for both analyte (DAS) and IS (ERL) from plasma samples. Recovery results were summarized in Table 3.

**Table 3:** Evaluation of the extraction efficiency and matrix effect for the determination of DAS in rat plasma by the proposed UPLC-MS/MS method.

Concentration spiked (ng/ml)	Mean recovery (%) ± RSD^a^	E_r_(%)
**Extraction efficiency**		
1	95.11±3.20	-4.55
3	96.39±3.20	-3.61
250	98.18±0.88	-1.82
450	95.49±5.40	-4.51
**Matrix effect**		
1	97.39±1.14	-2.61
3	96.94±3.35	-3.06
250	96.70±4.04	-3.30
450	94.34±3.64	-5.66

^a^Mean recovery (%) ± RSD of six determinations

E_r_%,Percentage relative error.

The matrix effect was determined using the same QC samples used for evaluating the extraction recovery and the calculated % error for DAS was found to be not more than 5.66% (Table 3). Also, the matrix effect for ERL (IS) was also determined at the concentration level specified in actual analysis and it was found to be 2.63%. Since the matrix effect was nearly negligible, trace analysis of DAS in plasma samples was possible using the proposed method.

#### Precision and accuracy

Precision, in terms of RSD, was assessed at the two levels, intra-day and inter-day and the calculated values were in the range (1.94–4.36%) and (1.53–6.50%) for intra-day, and inter-day, respectively. Method accuracy, in terms of relative errors, was also assessed at the intra-day and inter-day levels where the calculated values were in the range (-4.04-(-1.79) %) and (-3.03-(-0.17) %), respectively. Results for intra-day and inter-day precision and accuracy were summarized in Table 4. High degree of method precision and accuracy was proved since all calculated values of RSD and relative errors were less than 15% for concentrations other than LLOQ and 20% for LLOQ.

**Table 4:** Evaluation of the intra-day and inter-day accuracy and precision for the determination of DAS in rat plasma by the proposed UPLC-MS/MS method.

	Intra-day (n = 6)		Inter-day (n = 18)	
Concentration added (ng/mL)	Mean recovery (%) ±RSD^a^	E_r_(%)	Mean recovery (%) ±RSD ^a^	E_r_(%)
1	98.21±1.94	-1.79	97.88±1.78	-2.12
3	96.33±4.36	-3.67	96.97±1.53	-3.03
250	96.61±4.05	-3.39	97.21±2.11	-2.79
450	95.96±3.25	-4.04	99.83±6.50	-0.17

^a^Mean recovery (%) ± RSD of six determinations

E_r_%, Percentage relative error.

#### Dilution integrity

Dilution integrity was evaluated in order to investigate the effect of dilution of plasma samples containing very high DAS concentrations beyond the linearity range of the proposed method. Fold dilutions (1:2 and 1:5) of concentrated samples yielded acceptable recoveries with error values (RSD) not more than 2.65% (2.56), Table 5. Since the calculated values were within the acceptance level (± 15%) acceptance limits, this indicates the integrity of DAS for up to five fold dilution of concentrated plasma samples.

**Table 5:** Evaluation of the dilution integrity of DAS in rat plasma.

Concentration spiked (ng/mL)	Dilution fold	Mean recovery (%) ±RSD^a^	E_r_(%)
800	1:2	97.35±2.56	-2.65
	1:5	98.95±1.41	-1.05

^a^Mean recovery (%) ± RSD of six determinations

E_r_%, Percentage relative error.

#### Stability studies

Stability studies were assessed using plasma samples spiked at two different DAS concentrations, namely 3 and 450 ng/mL. The calculated recovery values for DAS, Table 6, were not less than 96.13% with RSD not more than 5.41 indicating high degree of sample stability under different handling and storage conditions as described under the experimental section. Furthermore, DAS solutions were found stable when kept at room temperature for 6 h and up to 3 months at the refrigerator (4°C). Stability studies carried out in this work yielded nearly the same results as those obtained from previous studies [29–31].

**Table 6:** Evaluation of the stability of DAS in rat plasma.

Stability	Concentration added (ng/mL)	Mean recovery (%) ±RSD^a^
Auto-sampler stability (10°C, 56 h)	3	97.30±5.41
	450	98.47±3.14
Short-term stability (25°C, 6 h)	3	96.13±2.85
	450	97.24±3.83
Long-term stability (-30°C, 30 days)	3	97.93±2.58
	450	97.26±0.81
Freeze-thaw stability (-30°C, 3 cycles)	3	101.43±1.22
	450	99.47±0.24
Refrigerator (4°C, 3 months)	3	99.89±1.76
	450	98.09±3.93

^**a**^Mean recovery (%) ± RSD of six determinations.

### Application to pharmacokinetic interaction studies

The wide use of nutraceuticals, with the strong belief of its benefits and wide safety margin, among cancerous patients, contribute to the problem of food-drug interactions. The bioavailability of DAS, like other TKIs, is mainly affected by the function of ABC transporters as well as the CYP3A metabolizing enzymes [4–6]. Among the most common nutraceuticals are curcumin, olive oil, and cocoa preparations. Giving their potent anticancer effect as well as their curative effect in UC and other pathogenic colitis, they could be widely used with DAS for their synergistic effect in cancerous patients, or those suffer from UC. Thus, it was extremely important to study if there would be any PK interaction when such nutraceuticals are given concomitantly with DAS. It was thought that studying the PK interaction on UC rats would resemble what could be found in actual clinical situation, thus yielding more realistic results compared to normal rats. UC was introduced in the working rats using acetic acid as described in previous literature [37]. The proposed UPLC -MS/MS method was used in this respect. As a control group, DAS was given to rats of group I at a dose of 25 mg/kg. Then further six groups of Wistar rats were administered combinations of DAS (25 mg/kg) with either curcumin (groups II and III), olive oil (groups IV, V), or cocoa preparations (groups VI, VII), in the same way as described under the experimental section. From each group, blood samples were separately withdrawn at pre-determined time intervals. Samples were treated and analyzed, and then the concentration of DAS found in each sample was calculated. Representative MRM chromatograms of rat plasma samples withdrawn 1 h after the concomitant administration of DAS with each of the studied preparations were shown in Fig 2. The mean DAS plasma concentration time profiles reported either alone or following concomitant administration of each of the studied preparations were given in Fig 3. Table 7 summarizes the different PK parameters calculated in each case, including maximum plasma concentration (C_max_), time to reach the maximum plasma concentration (t_max_), half-life (t_1/2_), the area under the curve from 0 to t (AUC_0-t_) and from 0 to ∞ (AUC_0-∞_), mean residence time from 0 to t (MRT_0-t_) and from 0 to ∞ (MRT_0-∞_), clearance (CL), and volume of distribution (V_d_). The significance of the differences in each of the calculated PK parameters between treated groups (II to VII) and the control group (group I) was assessed using Dunnett’s test. α values less than 0.05 were taken as the significance limit. Experimental data and the pharmacokinetic parameters were expressed as the mean±standard deviation. The obtained results showed that PK parameters of DAS, including C_max_, AUC_0-t_, AUC_0-∞_, CL, and V_d_ were not significantly different among each of the animal groups (II to VII), compared with the control group (I). Only differences in t_max_, t_1/2_, MRT_0-t_, and MRT_0-∞_ were noticed in some groups. These findings suggested that neither of the tested nutraceuticals caused significant changes in DAS bioavailability inspite of the inhibitory effect of curcumin/olive oil/cocoa extract on the function of P-gp transporters and/or CYP3A activity involved in DAS clearance [11, 13, 19]. During the study design, the effect of Bio-curcumin (rat group II) on the PK of DAS was predicted to be greater than that of regular curcumin preparation (rat group III) due to the enhanced absorption in the formulation of Bio-curcumin, compared with the regular one. Yet, the PK interaction between Bio-curcumin and DAS was still considered insignificant. Also, cocoa extracts caused a slight decrease in DAS bioavailability which could be attributed to gut-induced reduction in DAS absorption rather than an effect on its clearance, as previously reported with green tea extract co-administered with erlotinib and lapatinib [34, 38]. This unexpected behavior of the insignificant effect of the selected nutraceuticals on the bioavailability of DAS was similarly reported in a previous study dealing with the investigation of the PK effect of a well-known Chinese herbal medicine, Long-Dan-Xie-Gan-Tang formulation (LDXGT), and with two cytochrome P450 3A4 (CYP3A4) inhibitors, grapefruit juice and ketoconazole [39]. This study [39] concluded that co-administration of variable doses of the LDXGT formulation or grapefruit juice/ketoconazole did not cause a significant effect on the PK of sorafenib in rats. These implement to the mystery of “herb-drug interactions’ and the need of performing further clinical investigations to provide a clearer picture of the actual situation.

**Table 7:** Main pharmacokinetic parameters (mean±SD) after oral administration of DAS (25 mg/kg) to rats (*n* = 5).

	*Group I*	*Group II*	*Group III*	*Group IV*	*Group V*	*Group VI*	*Group VII*
	DAS	DAS + curcumin I	DAS + curcumin II	DAS + olive oil I	DAS + olive oil II	DAS + cocoa I	DAS + cocoa II
C_max_ (ng/mL)	230.48±30.97	292.52±47.10	245.89±28.15	292.21±56.63	264.15±76.34	221.15±74.54	164.39±32.76
t_max_ (h)	0.51±0.08	3.07±0.33*	1.02±0.43	2.02±0.49*	1.52±0.55	1.09±0.48	1.51±0.65
t_½_ (h)	17.89±2.15	11.56±1.12*	18.21±2.69	10.17±2.63*	7.97±1.03*	19.29±3.51	21.89±4.53
AUC_0-t_ (ng.h/mL)	1644.29±680.86	2170.53±694.07	1717.16±343.57	2045.52±783.38	1872.94±107.09	1428.03±313.85	1011.83±222.81
AUC_0-∞_ (ng.h/mL)	2658.39±595.97	3022.91±800.71	2751.22±800.01	2506.69±978.83	2143.86±692.91	2357.22±453.84	1988.69±263.76
MRT_0-t_ (h)	8.60±0.88	8.36±0.73	8.46±0.74	7.36±0.58	6.56±0.42*	8.59±1.25	9.16±0.85
MRT_0-∞_ (h)	24.39±4.29	17.47±4.70*	24.39±4.15	13.48±1.45*	10.19±0.74*	26.47±3.01	31.69±3.06*
CL (L/h/kg)	9.17±4.01	8.47±3.23	8.98±0.83	9.67±2.75	11.27±3.16	11.05±3.41	13.14±6.30
V_d_ (L/kg)	238.12±44.22	139.41±48.97	247.06±59.71	147.77±38.23	139.28±29.78	304.09±154.35	404.08±84.03

*Indicates significant difference using Dunnett’s test; critical value of 2.73 at α = 0.05, number of groups including control = 7, df = 28.

## Conclusion

In this study, UPLC -MS/MS method has been studied and validated for the purpose of measuring DAS in rat plasma samples. The proposed method has many advantages over the previously published LC-MS/MS methods for the determination of DAS [29–31] including LLOQ enabling the determination of very low concentrations required for terminal phase PK studies, shorter analysis time for high throughput analysis, and smaller injection volume, an important aspect where only small volumes of plasma samples are available e.g. pediatric and geriatric TDM. In addition, this method applies PPT/SPE as s superior clean-up combinational sample preparation technique providing improved sensitivity and selectivity, compared with PPT used in previous literature [29–31].

To our knowledge, this work was the first to investigate the effect of nutraceuticals, curcumin, olive oil, and cocoa extract, on the PK of co-administered DAS. Experimental study on rats revealed that co-administration of the selected nutraceuticals did not significantly alter the bioavailability of DAS in rats. These observations suggest the safety of their administration during the treatment protocols with DAS. However, further clinical investigations should be performed.

## Supporting information

S1 Appendix Data of plasma-concentration time profiles for all rat groups. (XLSX)
